# Deciphering Genomic Alterations in Colorectal Cancer through Transcriptional Subtype-Based Network Analysis

**DOI:** 10.1371/journal.pone.0079282

**Published:** 2013-11-15

**Authors:** Jing Zhu, Jing Wang, Zhiao Shi, Jeffrey L. Franklin, Natasha G. Deane, Robert J. Coffey, R. Daniel Beauchamp, Bing Zhang

**Affiliations:** 1 Department of Biomedical Informatics, Vanderbilt University, Nashville, Tennessee, United States of America; 2 Advanced Computing Center for Research and Education, Vanderbilt University, Nashville, Tennessee, United States of America; 3 Department of Electrical Engineering and Computer Science, Vanderbilt University, Nashville, Tennessee, United States of America; 4 Department of Medicine, Vanderbilt University, Nashville, Tennessee, United States of America; 5 Department of Cell and Developmental Biology, Vanderbilt University, Nashville, Tennessee, United States of America; 6 Departments of Surgery, Vanderbilt University, Nashville, Tennessee, United States of America; 7 Department of Radiology and Institute of Imaging Sciences, Vanderbilt University, Nashville, Tennessee, United States of America; 8 Department of Cancer Biology, Vanderbilt University, Nashville, Tennessee, United States of America; Ohio State University Medical Center, United States of America

## Abstract

Both transcriptional subtype and signaling network analyses have proved useful in cancer genomics research. However, these two approaches are usually applied in isolation in existing studies. We reason that deciphering genomic alterations based on cancer transcriptional subtypes may help reveal subtype-specific driver networks and provide insights for the development of personalized therapeutic strategies. In this study, we defined transcriptional subtypes for colorectal cancer (CRC) and identified driver networks/pathways for each subtype. Applying consensus clustering to a patient cohort with 1173 samples identified three transcriptional subtypes, which were validated in an independent cohort with 485 samples. The three subtypes were characterized by different transcriptional programs related to normal adult colon, early colon embryonic development, and epithelial mesenchymal transition, respectively. They also showed statistically different clinical outcomes. For each subtype, we mapped somatic mutation and copy number variation data onto an integrated signaling network and identified subtype-specific driver networks using a random walk-based strategy. We found that genomic alterations in the Wnt signaling pathway were common among all three subtypes; however, unique combinations of pathway alterations including Wnt, VEGF and Notch drove distinct molecular and clinical phenotypes in different CRC subtypes. Our results provide a coherent and integrated picture of human CRC that links genomic alterations to molecular and clinical consequences, and which provides insights for the development of personalized therapeutic strategies for different CRC subtypes.

## Introduction

Colorectal cancer (CRC) is a major cause of global cancer morbidity [1]. Over the past three decades, molecular genetic studies have revealed some critical mutations underlying the pathogenesis of CRC [2]. Recently, with the development of high-throughput sequencing technologies, thousands of genetic alterations have been identified in CRC. In addition to a limited number of well-known frequently-mutated oncogenes or tumor-suppressor genes such as APC, KRAS, PIK3CA and TP53, a much larger number of genes are mutated at a low frequency [3]. It has been suggested that somatic mutations found in cancers are either “drivers” or “passengers” [3]. How to distinguish drivers from passengers among thousands of low-frequency mutations has become a major challenge in cancer research.

Because signaling pathways and networks rather than individual genes govern the course of tumorigenesis and progression [4], several studies have used expert-curated pathways to help interpret high throughput genomic alterations [3], [5], [6]. Although helpful, these methods are limited by the coverage and completeness of curated pathways [7]. Consequently, network-based approaches such as HotNet [8] and NetWalker [9] have been developed, with successful application to the identification of subnetworks that are enriched with genomic variations [6], [10].

Network-based methods have started to provide a systems level understanding of complex genomic variations. However, because existing studies usually consider all tumor samples together in contrast to normal controls, they tend to identify signaling networks common to all tumor samples and may fail to address the heterogeneity among cancer genomes.

Transcriptional subtype analysis has provided great insights into disease biology, prognosis and personalized therapeutics for different cancer types [11], [12]. Interestingly, although both transcriptional subtype and signaling network analyses have proved useful in cancer genomics research, these two approaches are usually applied in isolation in existing studies. We reason that deciphering genomic alterations based on cancer transcriptional subtypes may help reveal subtype-specific driver networks and provide insights for the development of personalized therapeutic strategies.

For CRC, the TCGA (The Cancer Genome Atlas) network recently reported a classification of three transcriptional subtypes, which were named as “MSI/CIMP”, “Invasive”, and “CIN”, respectively [13]. However, the analysis is limited by several factors. First, the subtypes were identified from a relatively small patient cohort with only 220 samples and no independent validation was performed, leaving the generality of the subtype classification unproven. Next, due to the lack of survival data with enough follow up time for the TCGA cohort, clinical relevance of the subtypes remains to be established. It is not clear by which criteria the “invasive” subtype was labeled and whether it is supported by biological and clinical data. Moreover, although it is very interesting to link global genomic features such as Microsatellite Instability (MSI), CpG island methylation phenotype (CIMP), and chromosomal instability (CIN) with transcriptional subtypes, it remains a big challenge to translate these associations into targeted therapeutics for different CRC subtypes.

In this study, we hypothesize that highly heterogeneous genomic alterations observed in CRC may converge to a limited number of distinct mechanisms that drive unique gene expression patterns in different transcriptional subtypes. First, we extended the TCGA findings by performing subtype discovery based on gene expression data from 1173 CRC tumor samples accumulated during the past decade, validated identified subtypes in an independent cohort with 485 samples, and associated each subtype with unique biology and clinical outcome. Next, we mapped somatic mutation and copy number variation (CNV) data onto an integrated signaling network and identified a driver network for each subtype. The inferred networks and associated pathways correlated perfectly with downstream transcriptional programs characteristic for each subtype, providing strong circumstantial evidence for the effectiveness of our approach and the validity of our inference. Based on the unique combinations of pathway alterations and clinical outcomes, we have proposed specific therapeutic strategies for different CRC subtypes.

## Materials and Methods

### Data Acquisition and Processing

As shown in Table S1 in File S1, gene expression data for 1173 human CRC samples were downloaded from the Gene Expression Omnibus (GEO) database to build a discovery cohort. Gene expression data for an additional 485 human CRC samples were downloaded from the GEO database, the ArrayExpress Archive and The Cancer Genome Atlas (TCGA) to create a validation cohort. For each Affymetrix gene expression dataset, the Robust MultiChip Analysis (RMA) algorithm [14] was used for data processing, including quantile normalization and log2-transformation. To make the expression level comparable across datasets, we further normalized the expression level of each probe set in each sample relative to its average expression in all the samples in the same dataset, by subtracting its average in that dataset from each of its expression measurements [15]. As shown in Figure S1 in File S2, expression level across datasets is comparable after this normalization. Then, probe set identifiers were mapped to gene symbols based on the mapping file provided by corresponding databases. Probe sets mapped to multiple genes were eliminated. When multiple probe sets were mapped to the same gene, the median was used to represent the gene expression level. For TCGA gene expression data based on Agilent 244 K Gene Expression Microarray, Level 3 gene expression data (log2 lowess normalized (cy5/cy3) collapsed by gene symbol) were downloaded and the expression values for each gene were also mean centered. 10481 gene symbols common in all datasets were selected for the subsequent analyses.

To investigate gene expression changes in CRC samples relative to normal mucosa samples, gene expression data for these 182 samples were normalized together by the RMA algorithm [14]. Then, we normalized the expression level of gene g in each sample relative to its average expression in the five normal mucosa samples, by subtracting its average in the normal samples from each of its expression measurements.

To characterize the embryonic development of colon, we conducted a time course microarray study using the inbred C57BL/6 (Jackson Laboratories, Bar Harbor, ME) mice (Gene Expression Omnibus, GSE38831). This study was carried out in strict accordance with animal care and use guidelines and approval of the Vanderbilt Institutional Animal Care and Use Committee (IACUC). Mice were monitored throughout the experiment for signs of distress during their normal life cycle, although no experimental manipulations of these mice were carried out besides breeding. If signs of distress were seen during weekly monitoring, mice were euthanized by CO2 asphyxiation followed by cervical dislocation to reduce animal suffering. Seven samples corresponding to the mouse colonic development from E13.5 to E18.5 and adult (eight week post-natal) were collected. Embryonic colon collection and RNA preparation were performed as previously described [16]. RNA samples were submitted to the Vanderbilt Functional Genomics Shared Resource (FSGR, http://array.mc.vanderbilt.edu), where RNA was purified with the use of the RNeasy kit (QIAGEN, alencia, CA) and hybridized to the Affymetrix Mouse Genome 430 2.0 GeneChip Expression Arrays (Santa Clara, CA) according to manufacturer’s instructions. The RMA algorithm was used for data normalization. Mouse gene symbols were mapped to human gene symbols by the Human and Mouse Orthology list available from the Mouse Genome Informatics (http://www.informatics.jax.org/).

CNV data and somatic mutation data for TCGA samples with matched gene expression data were downloaded from the TCGA website.

Signaling pathways curated by NCI-Nature, Cancer Cell Map, and REACTOME were downloaded from the Pathway Commons database (latest version in Jun, 2011). BioCarta signaling pathways were downloaded from the NCI Pathway Interaction Database (Jun, 2011). Integrating pathways from all the above sources resulted in a signaling network containing 3152 genes and 47,833 edges. Its largest component contained 3078 genes and 47,772 edges, which was used for the inference of the upstream driver subnetworks.

### Co-expression Network and Module Analysis

**Figure 1:**
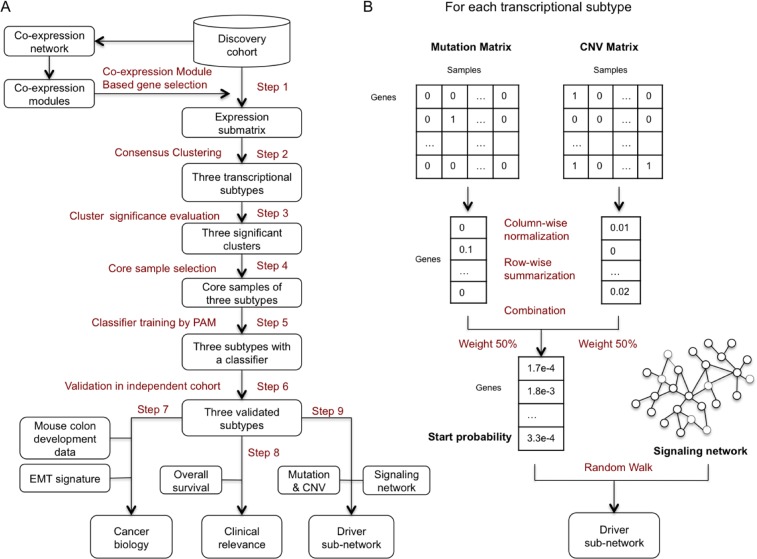
Schematic overview of methods used. (A) Study design. A detailed description of methods and data used in the study can be found in Table S2 in File S1; (B) Overview of the method used for inferring upstream driver subnetworks for individual subtypes.

Based on the gene expression matrix with 10,481 genes and 1173 samples for the discovery cohort, we calculated the Pearson’s correlation coefficients for all the 54,920,440 gene pairs. The construction of a co-expression network requires an appropriate selection of a threshold for the pair-wise correlation coefficients. To ensure the biological relevance of the constructed network, we used a knowledge-guided method for threshold selection [17]. Specifically, we evaluated functional similarity between each pair of genes based on the Gene Ontology (GO) biological process annotation using the Resnik’s semantic similarity [18]. The average functional similarities of gene pairs at various correlation ranges were calculated and plotted (Figure S2 in File S2). Based on the plot, the absolute Pearson’s correlation coefficient of 0.45 was selected for thresholding because a sharp increase in functional similarity occurs above this threshold for both positive and negative correlations. Based on the threshold above, a gene co-expression network with 8546 genes and 508,071 edges was constructed. We used our previously published Iterative Clique Enumeration (ICE) algorithm [17] to identify relatively independent co-expression modules from the gene co-expression network (Figure 1A and Table S2 in File S1). To focus on major transcriptional programs, we required each module to have at least 20 unique genes.

### Transcriptional Subtype Identification

For subtype discovery, we performed the consensus average linkage hierarchical clustering [19], based on genes in the above identified modules and all discovery samples (Figure 1A and Table S2 in File S1). The clustering was performed with GenePattern [20], using the same parameters as [12]. For the identified subgroups of CRC, SigClust was performed to evaluate the significance of all the pair-wise combinations [21] (Figure 1A and Table S2 in File S1). To identify samples that can’t represent its subgroup well, we evaluated how well each sample lies within its subgroup. Specifically, for sample *i*, we computed *a(i)* as the average distance between *i* and all other samples from the subgroup where *i* belongs. Then, the average distance between *i* and all samples from each of the other subgroups was computed respectively, and the smallest average distance, *b(i),* was identified. Next, we calculated the silhouette width *s(i)* as defined by: *s*(*i*) = (*b*(*i*) − *a*(*i*) )/max(*a*(*i*), *b*(*i*) ) [22]. Samples with a positive silhouette value were retained as “core” samples for the corresponding subtype (Figure 1A and Table S2 in File S1). This analysis was performed using the silhouette package in R.

### Construction of Subtype Classifier and Assigning Signature Genes for Each Subtype

We used a nearest shrunken centroid classification method, Prediction Analysis of Microarrays (PAM) [23] to build classifiers for the above defined subtypes. We ran 10-fold cross-validation 100 times to evaluate the performance of classifiers with different numbers of genes. For the selected classifier, we used the following rule to assign each gene in the classifier to a subtype. First, genes significantly up regulated (one-tail Student's t-Test, *p*<0.05) in one subtype compared to all other subtypes were defined as up-regulated genes for this subtype. Next, remaining genes that were significantly down regulated in one subtype compared to all other subtypes were defined as down-regulated genes for this subtype. For each subtype, both the up-regulated genes and the down-regulated genes were considered as signature genes.

### Driver Subnetwork Identification

We employed the Netwalker algorithm [9] for driver subnetwork identification (Figure 1A and Table S2 in File S1). Given the integrated signaling network and start probabilities for each node assigned based on the genomic variation status, the algorithm used the random walk with restart technique [24] to calculate a final priority score for each node based on the steady state probabilities. We set up the start probabilities for all 3078 genes based on their somatic mutation and CNV information for each subtype separately. As shown in Figure 1B, we computed two binary matrices based on the somatic mutation data (1 for non-silent mutation, 0 for others) and the CNV data (1 for genes within gains or losses regions with ratio ≥1.2 or ≤0.8, 0 for others) for each subtype separately.

To assign higher weight to genomic alterations observed in samples with fewer total number of alterations and alterations observed in multiple samples, we performed column-wise normalization followed by row-wise summarization for each binary matrix, and thus transformed each matrix into a vector. For a subtype, let’s denote *n* as the total number of genes and *m* as the total number of samples. The somatic mutation status of gene *i* is defined as:


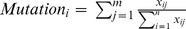
, where 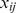 is the value for gene *i* in sample *j* in the somatic mutation matrix. Similarly, the CNV status of gene *i* is defined as: 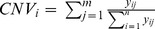, where 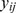 is the value for gene *i* in sample *j* in the CNV matrix. Next, 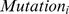 and 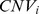 for each gene were combined together with equal weight. Start probability for gene *i* (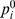) is thus defined as:
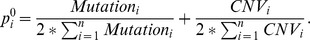



For the NetWalker algorithm, the restart probability was set to 0.5 and convergence was determined by 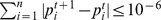, where 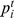 is the probability for gene *i* at the *t*th iteration.

To assess the statistical significance of the scores for each gene, we constructed 1000 sets of randomly permuted start probabilities and generated 1000 sets of random scores. For each gene in the network, a local *p* value was estimated by comparing the real score to random scores from the same gene, and a global *p* value was estimated by comparing the real score to random scores from all genes [9]. A significant global *p* value indicates the overall significance of the node with regard to the input start probabilities, while a significant local *p* value ensures that the significance is not simply due to network topology. For each subtype, the largest connected component formed by the significant genes (local *p*<0.05 and global *p*<0.05) was reported as the driver subnetwork.

### Survival Analysis

Standard Kaplan–Meier survival curves were generated for CRC subgroups, and the survival difference between groups was statistically evaluated using the log-rank test. The univariate and multivariate Cox proportional hazard regression analyses were used to evaluate potential independent prognostic factors associated with survival. All these analyses were performed using the survival package in R.

### GO and KEGG Pathways Enrichment Analysis

GO and KEGG pathway enrichment analyses were performed using WebGestalt, in which the hypergeometric test was used for enrichment analysis and the Benjamini-Hochberg procedure was used to control the False Discovery Rate (FDR) [25].

### Network Visualization

Networks were visualized using Cytoscape [26].

## Results

### Identification of Three Transcriptional Subtypes in CRC

We used a well-established method, Consensus Clustering [19], for the reliable identification of transcriptional subtypes [12], [27]. Usually, genes with high expression variance across a sample cohort are selected to cluster the samples [28]. This gene selection method is not able to distinguish biological variance from technical variance. Because the dysregulation of a key signaling pathway usually leads to coordinated expression changes for the downstream genes, groups of genes co-expressed across a sample cohort (i.e. co-expression modules) may better reflect underlying biological variance. Therefore, we first constructed a gene co-expression network and identified 33 co-expression modules with a total of 1472 unique genes from a discovery cohort with 1173 CRC samples (Table S1 in File S1). Then, we performed consensus clustering using genes from these modules, evaluated cluster significance and identified core samples for each cluster as previously described [12].

According to the consensus matrices and the empirical cumulative distribution function (CDF) plots in Figures S3A and S3B in File S2, the clustering stability increased considerably from 2 clusters to 3 clusters whereas no obvious increase was found for more than 3 clusters, suggesting that the 1173 CRC samples could be robustly divided into three clusters. We further evaluated cluster significance using SigClust [21] and confirmed statistical significance for all three clusters (Figure S3C in File S2). Following Verhaak et al. [12], we defined the “core samples” for each subtype as those with higher similarity to their own class than to any other classes and identified 985 core samples based on their positive silhouette width [22] (Figure S3D in File S2).

Next, we used PAM to build a classifier for the above defined subtypes. The shrinkage in PAM performs automatic gene selection and can potentially make the classifier more accurate by reducing the effect of noisy genes. The smallest average cross-validation error of 0.5% was achieved using all the 1472 genes based on 100 times of 10-fold cross validation, suggesting that noisy genes might have already been removed in our co-expression module-based gene selection procedure. With relaxed error rate requirement, PAM was able to further reduce the number of genes in the classifier. For example, when the error rate increased to 9%, a classifier with 853 genes was reported. Classifiers with reduced gene numbers are usually preferred in classification tasks; however, because an important goal in this study was to understand the biology underlying different subtypes, we selected the 1472-gene classifier to facilitate downstream GO enrichment analysis.

**Figure 2:**
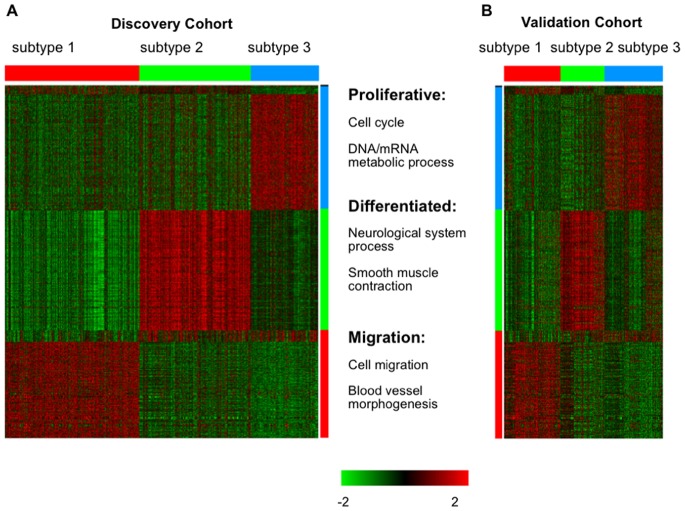
Identification and validation of three CRC subtypes based on gene expression data. (A) Using the 1472 selected genes, 985 core samples in the discovery cohort were clustered into three subtypes. For each subtype, samples and signature genes were labeled with same color (red bar for subtype 1, green bar for subtype 2 and blue bar for subtype 3). Biological processes enriched with signature genes for each subtype are shown beside the color bars; (B) Using the same ordering of signature genes and CRC subtypes as (A), the gene expression pattern for the 485 CRC samples from the validation cohort was shown.

Using the method described in Materials and Methods, we found 449 signature genes for subtype 1 (red bar in Figure 2, with 402 genes up-regulated and 47 genes down-regulated), 505 signature genes for subtype 2 (green bar in Figure 2, with 500 genes up-regulated and 5 genes down-regulated) and 512 signature genes for subtype 3 (blue bar in Figure 2, with 480 genes up-regulated and 32 genes down-regulated, Table S3 in File S3). Additionally, six genes that could not be defined as signature genes based on our criteria were labeled by the black bar in Figure 2 (at the top of the heat map).

To further test the biological relevance of the signature genes, we computed the pair-wise functional similarity for all genes in a signature based on the GO biological process annotation using the Resnik’s semantic similarity [18]. For each signature, the average pair-wise functional similarity of all signature genes was significantly higher than that of the same number of genes randomly selected from the 1472 genes (p<0.001 for subtype 1, p = 0.018 for subtype 2, and p = 0.001 for subtype 3, permutation test).

The small cross-validation error in the PAM analysis, distinctive expression patterns for each subtype as shown in Figure 2, and significant functional coherence of the signature genes for each subtype indicates that our CRC subtype classification is both accurate and well supported by distinct expression patterns of functionally related signature genes.

To compare our co-expression module-based approach for gene selection with the single gene-based method, we repeated the above clustering analysis based on the same number of genes (1472) with the largest median absolute deviation across the 1173 samples. Compared to our method, the single-gene based method generated larger average cross-validation error in the PAM analysis (2% vs 0.5%). Moreover, most of the subtype-specific signatures produced by the single-gene based method showed no significant functional coherence compared to random gene lists of the same size.

### Validation of the three CRC Subtypes in an Independent Cohort

To validate the CRC subtypes discovered above, we compiled an independent gene expression dataset with 485 CRC samples from six additional resources (Table S1 in File S1). The subtype labels of validation samples were predicted using the above constructed PAM classifier with the probabilities for individual samples provided in Table S4 in File S3. Using the same ordering of the genes and the CRC subtypes as those used in Figure 2A, gene expression for the 485 samples from the validation set was visualized in Figure 2B. A visual comparison between Figures 2A and 2B suggests that the three subtypes of CRC identified in the discovery set can be robustly rediscovered in the validation dataset.

### Direction of Gene Expression Changes

**Figure 3:**
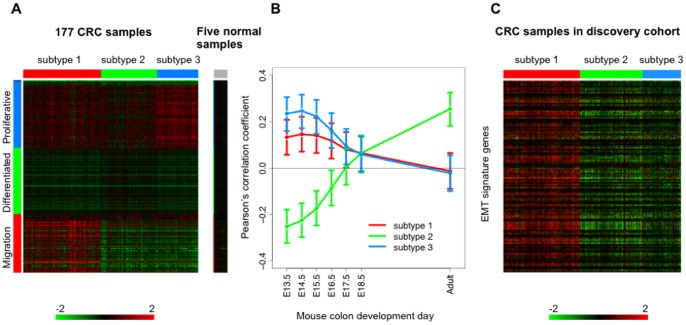
Unique cancer biology in three CRC subtypes. (A) Expression of signature genes in three CRC subtypes compared to expression in normal samples. The heat map was based on 1472 selected genes, and the gene expression dataset GSE17536 with 177 human CRC samples and five normal mucosal samples. (B) The correlation between the gene expression pattern of three CRC subtypes and the expression pattern of different stages of mouse colon development based on time related genes. The time series are indicated on the horizontal axis, while Pearson correlation coefficients are indicated on the vertical axis (Points represent Pearson correlation coefficients, bars represent 95% confidence intervals). (C) The expression of EMT signature genes in three CRC subtypes.

For subtype identification, we focused on the relative gene expression changes across all tumor samples. To further clarify the absolute direction of gene expression changes, we compared the expression of signature genes in each CRC subtype to their expression in normal colon mucosal samples. As shown in Figure 3A and Table S5 in File S1, in general, signature genes for subtype 1 were up-regulated in subtype 1 but down-regulated in subtype 2 and 3 compared to normal. Signature genes for subtype 2 were clearly down-regulated in subtypes 1 and 3 compared to normal, but the down-regulation was weaker in subtype 2. Signature genes for subtype 3 were up-regulated in all the CRC samples compared to normal, with the strongest up-regulation observed for subtype 3 and only moderate up-regulation observed for subtype 2. Similar trend was observed when comparing TCGA samples from the validation cohort with 22 normal samples from TCGA.

### Unique Cancer Biology for different CRC Subtypes

It has been suggested that CRC tumorigenesis and progression recapitulates embryonic development and epithelial mesenchymal transition (EMT) programs [29], [30]. To gain insight into the biological meaning of the three CRC subtypes, we investigated gene expression of the three subtypes within the contexts of normal colon development and EMT.

First, we generated a gene expression dataset (see Materials and Methods) of normal mouse colon development (E13.5–E18.5 and adult) and defined development-related genes as the top1000 genes with the largest median absolute deviation across different time points among those with a high correlation to developmental time points (absolute Spearman correlation coefficient >0.9). Based on the development-related genes, we evaluated the correlation between the expression patterns of different CRC subtypes and different developmental time points. Specifically, for each pair of CRC subtype and developmental time point, we calculated the Pearson’s correlation coefficient between the subtype centroids of the development-related genes and the expression levels of the same genes at the time point. As shown in Figure 3B, gene expression patterns of subtype 3 (blue line) were more similar to that of the early stage of mouse colon development whereas gene expression pattern of subtype 2 (green line) was more similar to that of the adult colon. Consistently, GO enrichment analysis showed that the subtype 3 signature was significantly enriched with genes in proliferation-related processes such as cell cycle (FDR = 9.95×10^−24^), DNA metabolic process (FDR = 9.18×10^−12^) and mRNA metabolic process (FDR = 2.63×10^−7^) (Figure 2). It is well known that early embryonic development is characterized by rapid cell proliferation. On the other hand, the subtype 2 signature was significantly enriched with genes involved in differentiated functions required for a more mature stage of development, such as smooth muscle contraction (FDR = 7.00×10^−4^) and neurological system process (FDR = 1.56×10^−14^). These genes are repressed in undifferentiated embryonic cells [31], which was in agreement with their markedly reduced expression in 3 but not subtype 2 (Figure 3A). Taken together, these results suggest that subtype 3 tumors reactivated the early colon developmental gene expression programs, whereas the subtype 2 tumors better maintained gene expression programs in normal adult colon.

Next, we examined the expression pattern of a previously published EMT signature [30] in these three subtypes. The signature was derived from a microarray dataset [30] comparing cell lines exhibiting a mesenchymal-like gene expression pattern (high levels of VIM and low levels of CDH1) vs. cell lines with an epithelial-like gene expression pattern (low levels of VIM and high levels of CDH1). 149 genes up-regulated in mesenchymal-like cell lines with a *p*-value <0.01 in *t*-test were used in our analysis. These genes had a much higher level of expression in subtype 1 tumors compared with the other two subtypes (Figure 3C). GO enrichment analysis showed that the subtype 1 signature was enriched with genes in cell migration (FDR = 2.0×10^−4^) and blood vessel morphogenesis (FDR = 7.49×10^−5^), biological processes closely related to EMT [32], [33]. Thus, the EMT program is characteristic of subtype 1. A complete list of GO terms enriched for the subtype signatures can be found in Table S6 in File S3.

### Distinct Clinical Outcomes for different CRC Subtypes

Using overall survival information available for samples from the Moffitt Cancer Center (GSE17536), the Vanderbilt Medical Center (GSE17537) and the Max Planck Institute (GSE12945), we performed a survival analysis to compare clinical outcomes for different CRC subtypes. Combining all the three datasets created a cohort with a total of 251 samples. This cohort included 161 samples from CRC stage II and III, where molecular stratification of these intermediate stage tumors could greatly facilitate treatment decision making [34].

**Figure 4:**
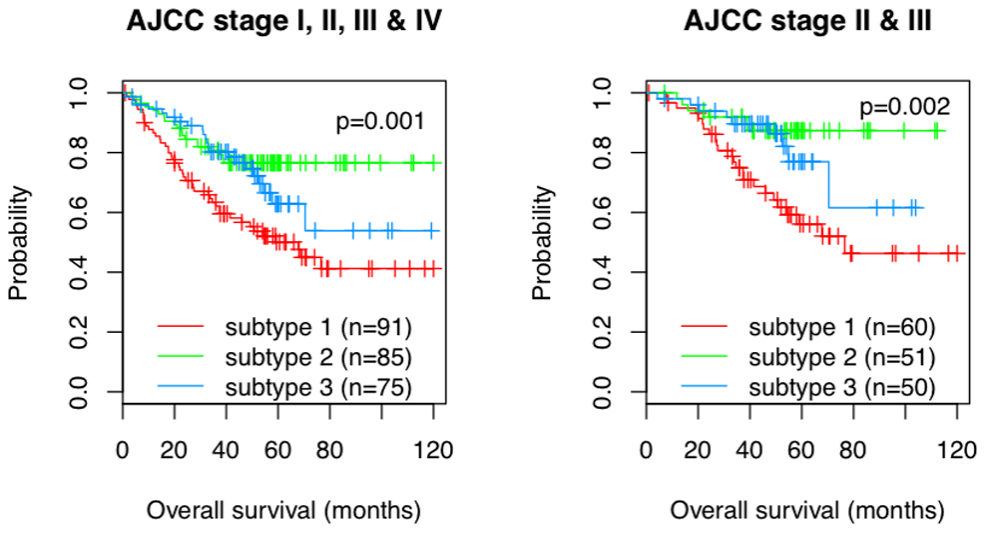
Kaplan-Meier plots of overall survival of patients from three CRC subtypes. Combining data from Moffit Cancer center, Vanderbilt Medical center and Max Planck Institute as a single cohort, we got 251 samples totally (left panel) and 161 samples from CRC stage II and III (right panel).

As depicted in Figure 4, the three subtypes showed significantly different outcomes (*p* = 0.001 for all the patients and *p* = 0.002 for stage II and III patients only, log-rank tests with H_0_: hazard_subtype1 =  hazard_subtype2 =  hazard_subtype3). Subtype 1 with a highly activated EMT program had the poorest overall survival among the three subtypes (5 yr survival rate = 50%), which was in agreement with previous reports on the association between EMT and CRC patient survival [30]. On the other hand, subtype 2, which more closely maintained the normal adult colon gene expression programs, had the best overall survival (5 yr survival rate = 77%). Interestingly, overall survival for subtype 3, with highly activated early colon developmental gene expression programs, was very similar to subtype 2 before year 4, but the survival rate dropped after year 4 (5 yr survival rate = 63%).

To evaluate the prognostic value of the subtype classification in combination with clinical variables including patient age at diagnosis, gender and AJCC stage, we performed univariate and multivariate Cox proportional hazards regression analyses based on the cohort of 251 samples (Table S7 in File S1). In the univariate analysis, both AJCC stage and the subtype classification were significantly associated with survival (p<0.05). In the multivariate analysis, the subtype classification still maintained the significance (*p* = 0.0041 for subtype 2 vs 1 and *p* = 0.036 for subtype 3 vs 1). Thus, the prognostic value of the subtype classification is independent of the AJCC stage.

### Comparison with the TCGA Subtype Classification

We compared our subtype classification with the TCGA classification [13] based on the 154 samples that were included in both studies. As shown in Table 1, we found a moderate but statistically significant overlap between two TCGA subtypes and our subtypes. Specifically, our subtype 1 and 2 showed enriched overlap with the TCGA subtypes “MSI/CIMP” and “Invasive”, respectively. We also compared our subtypes with the genomic information provided in the TCGA paper. As shown in Table 2, our subtype 1 was significantly enriched with CIMP-high and MSI-high tumors. Since MSI-high status has been linked to better overall survival, it is counterintuitive that our subtype 1 with poor overall survival was enriched with MSI-high tumors. However, we found that among the 19 MSI-high tumors in subtype 1, 12 tumors also have BRAF mutations. BRAF mutations have been were associated with worse overall survival [35], [36]. It was reported that among the MSI/BRAF-wild-type, MSI/BRAF-mutant, MSS/BRAF-wild-type and MSS/BRAF-mutant groups, MSI/BRAF-wild-type group had a significantly improved overall survival compared with all others. The remaining three groups had very similar survival outcomes [37]. After removing tumors with BRAF mutations, subtype 1 was not enriched with MSI-high tumors.

**Table 1:** Overlap between our subtypes and TCGA subtypes.

	MSI/CIMP 58 (38%)	Invasive 37 (25%)	CIN 56 (37%)	Total 151
Subtype 1	40 (59%,1.55x)**	10 (15%)	18 (26%)	68
Subtype 2	5 (16%)	14 (44%,1.76x)*	13 (41%)	32
Subtype 3	13 (25%)	13 (25%)	25 (49%)	51

Note: p values are computed by hypergeometric test and FDR (BH) was used for multiple test correction *: 0.01≤FDR<0.05; **: FDR<0.01.

**Table 2:** Overlap between our subtypes and TCGA CIMP_H, MSI and Hypermuated annotations.

	Methylation Available	CIMP_H	MSI Available	MSI_H	Mutation Available	Hyper_Mutated
Subtype 1	68 (44%)	19 (66%)*	68 (44%)	19 (68%)*	62 (44%)	18 (62%)
Subtype 2	33 (22%)	4 (14%)	33 (21%)	3 (11%)	30 (21%)	3 (10%)
Subtype 3	52 (34%)	6 (21%)	53 (34%)	6 (21%)	50 (35%)	8 (28%)
Total	153	29	154	28	142	29

Note: p values are computed by hypergeometric test and FDR (BH) was used for multiple test correction *: 0.01≤FDR<0.05.

During our manuscript preparation, two alternative CRC subtype classification schemes have been proposed and applied to the TCGA samples [38], [39]. As shown in Table S8 and Table S9 in File S1, our subtype 1 overlapped with the subtypes with poor prognosis in these classification schemes (i.e. the stem-like subtype and the CCS3 subtype), whereas our subtype 2 overlapped with the subtypes with good prognosis (i.e. the Goblet-like subtype and the CCS1 subtype). Therefore, an overall consistency was found for the three classification schemes.

A contradictory observation was that the TCGA invasive subtype showed the highest overlap with our subtype 2, which was found to have the best clinical outcome among the three subtypes (Figure 4). We further investigated the TCGA subtypes within the context of EMT and embryonic development. Although the pattern was not as clear as in our study (Figure S4 left in File S2), the EMT signature genes were relatively up-regulated in the MSI/CIMP subtype whereas down-regulated in the invasive subtype (Figure S4 right in File S2), linking the invasive subtype to potentially better clinical outcome based on previously established association between EMT and patient survival [30]. Moreover, the gene expression pattern of the invasive subtype showed the highest similarity to that of the adult colon (Figure S5, right in File S2), suggesting that the TCGA naming of this subtype is misleading.

### Inferring Upstream Driver Subnetworks for different CRC Subtypes

Based on the hypothesis that highly heterogeneous genomic alterations of CRC may converge to a limited number of distinct mechanisms that drive unique gene expression patterns in different CRC subtypes, we attempted to elucidate these distinct mechanisms by inferring upstream driver subnetworks for the unique gene expression patterns and their associated tumor physiologies in different CRC subtypes.

This analysis was based on a subset of The Cancer Genome Atlas (TCGA) samples from the validation dataset that had matched somatic mutation and CNV data (see Materials and Methods). After filtering for samples with both somatic mutation and CNV information, we identified 30 subtype 1 samples, 15 subtype 2 samples, and 22 subtype 3 samples.

To infer upstream driver subnetworks that are enriched with CNVs and/or somatic mutations, we used the Netwalker algorithm [9] as described in Materials and Methods. To set the start probability for the 3078 nodes in the signaling network for each subtype (Figure 1B), we first computed two binary matrices based on the somatic mutation data (1 for non-silent mutation, 0 for others) and the CNV data (1 for genes within gains or losses regions with ratio ≥1.2 or ≤0.8 [40], 0 for others), respectively. To assign higher weight to alterations observed in samples with fewer total number of alterations and alterations observed in multiple samples, we performed column-wise normalization followed by row-wise summarization for each binary matrix, and thus transformed each matrix into a vector (see Materials and Methods). Finally, the two vectors indicating the somatic mutation and CNV status of each gene were combined together with equal weight.

Using the genomic variation status for each gene as the start probability, we performed the Netwalker analysis and identified three driver subnetworks constituted of genes with significant priority scores (local *p*<0.05 and global *p*<0.05, see methods) for subtypes 1, 2 and 3, respectively (Figure S6 in File S2). The driver subnetwork for subtype 1 had 121 nodes and 373 edges; the one for subtype 2 had 107 nodes and 307 edges; and the one for subtype 3 had 101 nodes and 196 edges. As shown in Figure S7 in File S2, the driver subnetworks had limited number of overlapping nodes, indicating possibly distinct underlying mechanisms.

### Pathways Associated with Subtype-specific Networks

To associate these driver subnetworks with known pathways, we performed functional enrichment analysis based on the signaling pathways curated in the KEGG database (Table S10 in File S3). Consistent with our current understanding of CRC, the Wnt signaling pathway genes were enriched in all three driver subnetworks, although at different levels of significance (FDR = 1.56×10^−6^, 0.013, and 2.0×10^−4^ for subtypes 1, 2 and 3, respectively). In addition, the driver subnetwork for subtype 1 was enriched with genes in the VEGF signaling pathway (FDR = 1.0×10^−4^), and that for subtype 2 was enriched with genes in the Notch signaling pathway (FDR = 1.7×10^−3^). We further performed the Fisher’s exact test to examine the subtype-specificity of the VEGF and NOTCH pathways. The results showed that genes from the VEGF pathway were significantly over-represented in the driver subnetwork specific to subtype 1 compared to the other two subtypes (p = 0.028, Fisher’s exact test) and genes from the NOTCH pathway were significantly over-represented in the driver subnetwork specific to subtype 2 (p = 0.019, Fisher’s exact test).

Because our approach cannot distinguish activating mutations from deactivating mutations, we checked the expression change of some critical genes in the identified signaling pathways using the gene expression dataset GSE17536 with 177 CRC samples and five normal samples. We found that CTNNB1, DKK2, LEF1, LRP5, MYC and RUVBL1 were all up-regulated in all three subtypes compared to normal samples (one sided student’s t tests, *p*<0.02), suggesting an active status of the WNT signaling in all three subtypes. PIK3CA was only up-regulated in subtype 1 (one sided student’s t tests, *p* = 0.037), suggesting an active status of the VEGF signaling in subtype 1. NOTCH1 was up-regulated in all three subtypes compared to normal samples (one sided student’s t tests, *p* = 2.1×10^−4^, 4.2×10^−5^ and 2.0×10^−5^), suggesting an active status of Notch signaling in all three subtypes. However, RBPJ was only up-regulated in subtypes 1 and 3 (one sided student’s t test, *p* = 9.4×10^−6^ and 9.6×10^−4^), suggesting a relatively less-active status of Notch signaling in subtype 2 compared to the other two subtypes.

### Evaluation of Inferred Signaling Pathways

A well-recognized challenge in computational network and pathway inference is the lack of gold standard for an objective evaluation. In this study, because subtype-specific gene expression signatures and driver networks and pathways were independently identified from gene expression data and genomic data respectively, we assessed our pathway inference by checking the consistency between the inferred pathways and observed gene expression changes for each subtype. Correlating upstream genomic alterations to downstream transcriptional changes also allowed us to further assess whether the genomic alterations had resulted in pathway activation or inhibition. The c-Myc (MYC) transcription factor is a well-known downstream effector of the Wnt signaling pathway [41], whose activation induces the expression of cell proliferation genes [42]. The Wnt signaling pathway was inferred for all three subtypes. Consistently, genes involved in cell proliferation were up-regulated in all three subtypes compared to normal colon (Figure 3A), suggesting an activation of the Wnt signaling pathway in all three subtypes. As a more direct evidence, the signature genes for subtype 3 were significantly enriched with direct targets of c-Myc (*p* = 0.006, hypergeometric test), where the c-Myc target list were downloaded from the MsigDB (version 2.5). The VEGF signaling pathway was inferred specifically for subtype 1. It has been reported that alterations in the VEGF signaling pathway among Wnt-activated cells may activate the expression of genes involved in cell migration, angiogenesis, and the EMT program [32], [43], [44], [45], which was consistent with the specific up-regulation of these genes in subtype 1 (Figure 3A) and suggested an activation of the VEGF pathway in this subtype. Genes suppressed by Notch signaling, as exemplified by the transcription factor ATOH1 that promotes intestinal stem cell differentiation toward secretory lineages [46] and those involved in neurological system process (such as HTR1B, HTR6 and NLGN3), were highly repressed in subtypes 1 and 3 as compared to normal colon (Figure 3A), suggesting an active status of the Notch signaling in these subtypes. Inhibition of the Notch signaling pathway among Wnt-activated cells may lead to cell cycle exit and cell differentiation [42]. Based on the relatively higher-level expression of genes involved in differentiated functions in subtype 2 (Figure 2, Figure 3A), genomic alterations in the Notch signaling pathway may have resulted in reduced pathway activity in this subtype. These results are consistent with the above described expression changes for critical genes in the signaling pathways, and they suggested that inferred pathways correlated well with the downstream gene expression patterns for each subtype, thus providing strong evidence to support the validity of our inference.

### Pathway Landscape of Genomic Alterations in CRC

**Figure 5:**
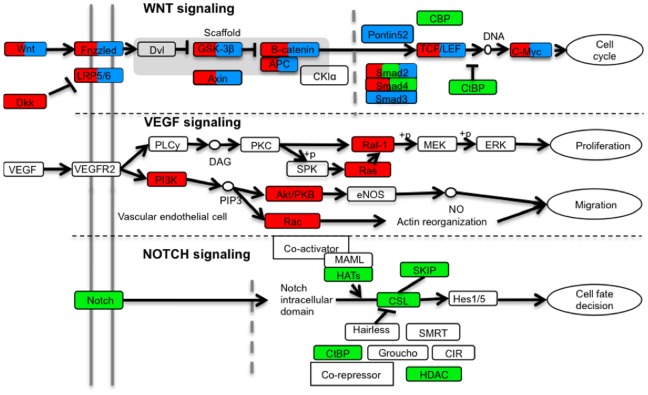
Signaling pathways enriched with upstream driver subnetworks for three CRC subtypes. Wnt, VEFG and Notch signaling pathways from KEGG were simplified. Genes with genomic alterations in subtype 1, 2 and 3 were colored with red, green and blue, separately. Genes with genomic alterations in more than one subtype were given multiple colors.

Figure 5 depicts a pathway landscape for genomic alterations in CRC, with alterations observed in different subtypes indicated by different colors (red for subtype 1, green for subtype 2 and blue for subtype 3). For genes in the landscape, the normalized values indicating their somatic mutation and CNV status in individual samples were visualized in the heat maps in Figure S8 in File S2. From these figures, it is obvious that only a few genes (e.g. APC, TP53, KRAS) were mutated in multiple samples, whereas most of the genes were mutated in only one sample. Moreover, although alterations in the Wnt signaling pathway were observed in samples from all subtypes, other pathway alterations were clearly subtype-specific. Thus, our transcriptional subtype-based network analysis approach provides an effective means for deciphering complex genomic alterations that cannot be easily interpreted at individual gene level or without subtype stratification.

## Discussion

Rapid advancement in high-throughput sequencing technologies has shifted our focus from data acquisition to data interpretation. For cancer genomic studies, pinpointing the genomic alterations underlying tumor initiation and progression and determining their downstream functional effects are among the most critical and challenging questions. In this study, we have developed a novel transcriptional subtype-based network inference strategy for deciphering genomic alterations. Many existing studies performed pathway and/or network analysis in tumor cohorts without considering transcriptional subtype classification [6], [13]. They tend to identify signaling pathways or networks common to all tumor samples and may fail to address the heterogeneity among cancer subtypes. Some studies have associated known cancer genes or cancer related pathways with subtypes [12], [47]; however, they are limited by the coverage and completeness of known cancer genes and pathways. We inferred upstream driver signaling subnetworks for different CRC subtypes. In addition to identifying genes annotated in known cancer related pathways, these networks provide novel subtype-specific candidates for future investigation.

Both the TCGA and our studies have identified three CRC subtypes, and a moderate but statistically significant overlap between two TCGA subtypes and our subtypes was observed (Table 1). A careful comparison between the two classifications has revealed some important information that could inform future tumor subtype studies. (i) Subtype analysis can benefit from increased sample size and improved gene selection methods. In the TCGA classification, tumors in the same subtype demonstrated somewhat inconsistent expression patterns even for the discovery cohort (Supplementary Figure 2 in [13]). In contrast, consistent expression patterns were found for tumors in the same subtype for both the discovery and validation cohorts in our study (Figure 2), which may be attributed to the large sample size (1173 samples in our discovery cohort *vs* 220 samples in the TCGA study) and our module-based gene selection method. (ii) Subtype characterization requires associating the subtypes with clinical outcome and known cancer biology. The TCGA study was limited by the lack of survival data with enough follow up time for establishing clinical relevance of the subtypes and according to our analysis, the naming of the invasive subtype in the TCGA classification is misleading. (iii) Subtype analysis can be strengthened by associating the subtypes with genomic data. The TCGA study linked genomic features such as MSI/CIMP, hypermutation, and CIN with the transcriptional subtypes, and we also found statistically significant enrichment of CIMP-high tumors in our subtype 1. These are interesting findings, however, it remains unclear how these global genomic features drive downstream distinct expression patterns in different transcriptional subtypes. Our transcriptional subtype-based signaling network analysis provides a more direct means to link genomic alterations to subtype-specific gene expression changes. The TCGA paper also identified frequently altered signaling pathways in CRC, however, these alterations were not associated with transcriptional subtypes.

Table 3 summarizes our findings of driver pathways, transcriptional programs, biological processes and clinical outcomes associated with each of the three subtypes. Taken together, they provide a coherent and integrated picture of human CRC that links genomic alterations to molecular and clinical consequences. Not surprisingly, genomic alterations in the Wnt signaling pathway were common among all three subtypes, consistent with its critical role in CRC initiation. However, it is the combination of pathway alterations that drove distinct molecular and clinical phenotypes in different CRC subtypes. Activation of both Wnt and Notch in subtypes 1 and 3 may lead to increased proliferation without differentiation [42], which explains the similarity between the two subtypes in the context of proliferation and differentiation (Figure 3A, the top two blocks of genes) and the activation of early colon embryonic development programs in these subtypes (Figure 3B). For subtype 2, genomic alterations in the Notch signaling pathway may keep the pathway in a less active status and lead to cell cycle exit and cell differentiation [42]. Therefore, alterations in the Notch signaling pathway may help subtype 2 maintain a transcriptional program similar to that of normal adult colon (Figure 3B), and thus a favorable clinical outcome. On the other hand, subtype 1 distinguishes itself from the other two subtypes by the activation of the cell migration (Figure 3A, the bottom block of genes) and EMT (Figure 3C) programs, which may contribute directly to the poor clinical outcome of this subtype and can be associated with genomic alterations in the VEGF signaling pathway [32], [43], [44], [45].

**Table 3:** Distinct mechanisms driving unique cancer biology and clinical outcome in different transcriptional subtypes.

	Driver pathway			Transcriptional program	Biological theme	5yr survival rate	
	Wnt	VEGF	Notch			Stage I–IV	Stage II–III
Subtype 1	**	**		EMT	Cell Migration	50%	56%
Subtype 2	*		**	Normal adult colon	Differentiated	76%	87%
Subtype 3	**			Early colon embryonic development	Proliferative	63%	77%

Note: **: FDR<0.01; *: 0.01<FDR<0.05; EMT: epithelial mesenchymal transition.

In addition to a more comprehensive understanding of human CRC, our findings also provide guidance on possible personalized therapeutic strategies for different subtypes. The poor outcome for subtype 1 patients calls for an emphasis on the development of efficient therapeutic strategies for this subtype, and targeting the VEGF pathway or simultaneously targeting the VEGF and Notch pathways seem like rational choices. Interestingly, clinical trials on the anti-VEGF therapy for colorectal cancer patients have reached inconsistent conclusions [48], [49], [50], [51]. Our results strongly argue for the integration of patient stratification into future trials. For instance, subtype 1 patients may be more sensitive to the anti-VEGF therapy because of the highly activated VEGF signaling, whereas other patients might not benefit from this therapy. For subtype 3 patients, Notch inhibitors might prove to be helpful. Based on the higher observed 5-year survival rate for subtype 2, these patients may not benefit as much from adjuvant chemotherapy after surgery in comparison to other subtypes. Genes in the annotated pathways shown in Figure 5 may serve as candidates for targeted treatment. In addition, genes in the networks shown in Figure S6 in File S2 but not included in the annotated pathways, may serve as novel candidates for targeting.

To test the possible targeted therapeutic strategies, we also tried to identify cell line models for the subtype by applying our classifier to CRC cell lines with publicly available gene expression data. However, representative cell line models were not identified for subtype 1 with the poor overall survial, possibly due to the lack of highly expressed cell migration and blood vessel morphogenesis signatures related to EMT program in CRC cell lines. We are working on the development and use of xenograft models for this subtype.

## Supporting Information

File S1 **This file contains Tables S1, S2, S5, S7, S8 and S9.**
(DOCX)

File S2 **This file contains all supplementary figures (S1–S8).**
(PDF)

File S3 **This file contains Tables S3, S4, S6, and S10.**
(XLSX)
