# Supplementary material for: Deciphering Genomic Alterations in Colorectal Cancer through Transcriptional Subtype-Based Network Analysis
Source: PLoS One. 2013 Nov 15;8(11):e79282. doi: 10.1371/journal.pone.0079282 (PMC3829853; doi:10.1371/journal.pone.0079282)
Supplement: File S1 — This file contains Tables S1, S2, S5, S7, S8 and S9. (DOCX) [file pone.0079282.s001.docx]

**Supplementary Tables**

**Table S1. Resources of gene expression data**

| Data ID | Sample size | Platform | Overall Survival available |
| --- | --- | --- | --- |
|  | Discovery cohort | |  |
| GSE14333_aus | 101 | U133_Plus_2 |  |
| GSE17536 | 177 | U133_Plus_2 | Yes |
| GSE17537 | 55 | U133_Plus_2 | Yes |
| GSE2109 | 292 | U133_Plus_2 |  |
| GSE13294 | 155 | U133_Plus_2 |  |
| GSE18088 | 53 | U133_Plus_2 |  |
| GSE26682 | 176 | U133_Plus_2 |  |
| GSE26906 | 90 | U133_Plus_2 |  |
| GSE13067 | 74 | U133_plus_2 |  |
|  | Validation cohort | |  |
| GSE16125 | 36 | Human Exon 1.0 ST |  |
| GSE24551 | 160 | Human Exon 1.0 ST |  |
| GSE12945 | 62 | HU133 | Yes |
| GSE4045 | 37 | HU133 |  |
| MEXP383 | 36 | HU133 |  |
| TCGA | 154 | Agilent |  |

**Table S2. Summary of methods and datasets used in the study**

| **Steps** | **Purpose** | **Methods and software** | **Datasets** |
| --- | --- | --- | --- |
| 1 | Gene selection before clustering | A gene co-expression network was first constructed based on Pearson’s correlation and then was divided into co-expression modules based on Iterative Clique Enumeration (ICE) algorithm [[1](#_ENREF_1)]. Genes from these modules were used for consensus clustering. | The discovery cohort with 1173 CRC samples. |
| 2 | Subtype identification | Consensus average linkage hierarchical clustering performed with “GenePattern” [[2](#_ENREF_2)]. | The discovery cohort with 1173 CRC samples. |
| 3 | Cluster significance evaluation | R package “SigClust” [[3](#_ENREF_3)] was used to evaluate the significance of all the pair-wise combinations of identified clusters. | The discovery cohort with 1173 CRC samples. |
| 4 | Core sample selection | R package “silhouette” [[4](#_ENREF_4)] was used to evaluate how well each sample lies within its subgroup. Only samples that can represent its subgroup well were left as core samples. | The discovery cohort with 1173 CRC samples. |
| 5 | Classifier training | A nearest shrunken centroid classification method, Prediction Analysis of Microarrays (PAM) [[5](#_ENREF_5)] implemented in R package “pamr” was used to build a classifier for the above defined subtypes. | The discovery cohort with 1173 CRC samples. |
| 6 | Validation in independent cohort | The subtype labels of validation samples were predicted using the above constructed PAM classifier. | The validation cohort with 485 CRC samples. |
| 7 | Identifying unique cancer biology for different CRC subtypes | The gene expression of the three subtypes was investigated in the contexts of normal colon development and EMT. | Gene expression dataset of normal mouse colon development (GSE38831). A previously published EMT signature [[6](#_ENREF_6)]. |
| 8 | Comparing the clinical outcomes of different CRC subtypes | R package “survival” was used to generate Standard Kaplan–Meier survival curves, evaluate the survival difference between groups using the log-rank test and perform the univariate and multivariate Cox proportional hazard regression analyses. | Overall survival information available for samples from the Moffitt Cancer Center (GSE17536), the Vanderbilt Medical Center (GSE17537) and the Max Planck Institute (GSE12945). |
| 9 | Inferring upstream driver subnetworks for different CRC subtypes | The Netwalker algorithm [[7](#_ENREF_7)] was employed for driver subnetwork identification. | CNV data and somatic mutation data from TCGA samples. A signaling network, containing 3152 genes and 47,833 edges, was constructed by integrating signaling pathways from public databases. |

Reference:

1. Shi, Z., C.K. Derow, and B. Zhang, Co-expression module analysis reveals biological processes, genomic gain, and regulatory mechanisms associated with breast cancer progression. BMC Systems Biology, 2010. **4**: p. 74.

2. Reich, M., et al., GenePattern 2.0. Nat Genet, 2006. **38**(5): p. 500-1.

3. Liu, Y., et al., Statistical significance of clustering for high dimension low sample size data. Journal of the American Statistical Association, 2008. **103**: p. 1281-1293.

4. Rousseeuw, P.J., Silhouettes: A graphical aid to the interpretation and validation of cluster analysis. Journal of Computational and Applied Mathematics, 1987. **20**: p. 53-65.

5. Tibshirani, R., et al., Diagnosis of multiple cancer types by shrunken centroids of gene expression. Proc Natl Acad Sci U S A, 2002. **99**(10): p. 6567-72.

6. Loboda, A., et al., EMT is the dominant program in human colon cancer. BMC Med Genomics, 2011. **4**: p. 9.

7. Zhang, B., et al., Relating protein adduction to gene expression changes: a systems approach. Mol Biosyst, 2011. **7**(7): p. 2118-27.

**Table S5 Signature genes significantly up- or down-regulated in each subtype compared to the normal tissue**

|  | Subtype 1 (red) | Subtype 2 (green) | Subtype 3 (blue) |
| --- | --- | --- | --- |
| 480 genes relatively up in Subtype 3 (blue) | 318 up  43 down  119 NS | 229 up  88 down  163 NS | 418 up  12 down  50 NS |
| 500 genes relatively up in Subtype 2 (green) | 55 up  322 down  123 NS | 101 up  189 down  210 NS | 60 up  315 down  125 NS |
| 402 genes relatively up in Subtype 1 (red) | 211 up  67 down  124 NS | 68 up  230 down  104 NS | 73 up  237 down  92 NS |

NS: not significant (p > 0.05 in one-tailed student’s t-test)

**Table S7 Univariate and multivariate Cox proportional hazard regression analyses of overall survival in 251 CRC samples**

|  | Univariate | |  | Multivariate | |
| --- | --- | --- | --- | --- | --- |
|  | HR (95% CI) | p value |  | HR (95% CI) | p value |
| Age | 1.01 (0.99-1.03) | 0.33 |  | 1.02 (1.00-1.04) | 0.033 |
| Gender (M or F) | 1.05 (0.69-1.61) | 0.81 |  | 0.97 (0.62-1.52) | 0.91 |
| AJCC stage (I, II, III, IV) | 2.98 (2.26-3.93) | 1.21e-14 |  | 3.11 (2.33-4.12) | 7.55e-15 |
| Subtype (2 vs 1) | 0.40 (0.23-0.69) | 8.50e-4 |  | 0.45 (0.26-0.78) | 0.0041 |
| Subtype (3 vs 1) | 0.55 (0.33-0.92) | 0.024 |  | 0.58 (0.34-0.97) | 0.036 |

**Table S8 Overlap between our subtypes and Sadanandam, et al.’s subtypes**

|  | Enterocyte/  Goblet-like (42) | Inflammatory (42) | Stem-like (46) | TA (85) | Total |
| --- | --- | --- | --- | --- | --- |
| Subtype 1 | 8 | 31** | 38** | 21 | 98 |
| Subtype 2 | 18** | 5 | 4 | 18 | 45 |
| Subtype 3 | 16 | 6 | 4 | 46** | 72 |

Note: p values are computed by hypergeometric test and FDR (BH) was used for multiple test correction **: FDR<0.01

**Table S9 Overlap between our subtypes and De Sousa, et al. ’s subtypes**

|  | CCS1 (126) | CCS2 (34) | CCS3 (55) | Total |
| --- | --- | --- | --- | --- |
| Subtype 1 | 31 | 18 | 49** | 98 |
| Subtype 2 | 38** | 5 | 2 | 45 |
| Subtype 3 | 57** | 11 | 4 | 72 |

Note: p values are computed by hypergeometric test and FDR (BH) was used for multiple test correction **: FDR<0.01
