# Supplementary material for: Deciphering Genomic Alterations in Colorectal Cancer through Transcriptional Subtype-Based Network Analysis
Source: PLoS One. 2013 Nov 15;8(11):e79282. doi: 10.1371/journal.pone.0079282 (PMC3829853; doi:10.1371/journal.pone.0079282)
Supplement: File S2 — This file contains all supplementary figures (S1–S8). (PDF) [file pone.0079282.s002.pdf]

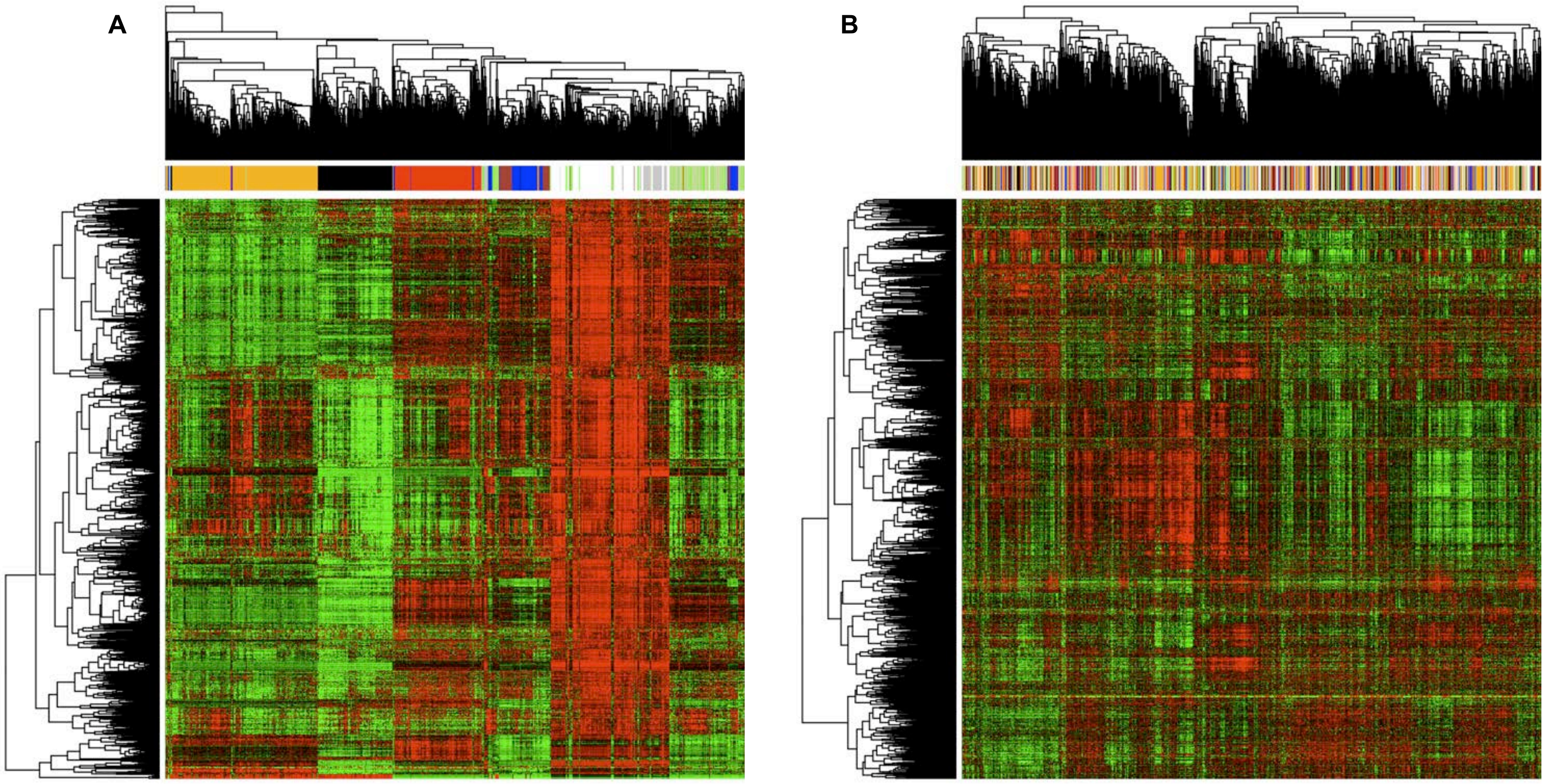

Figure S1

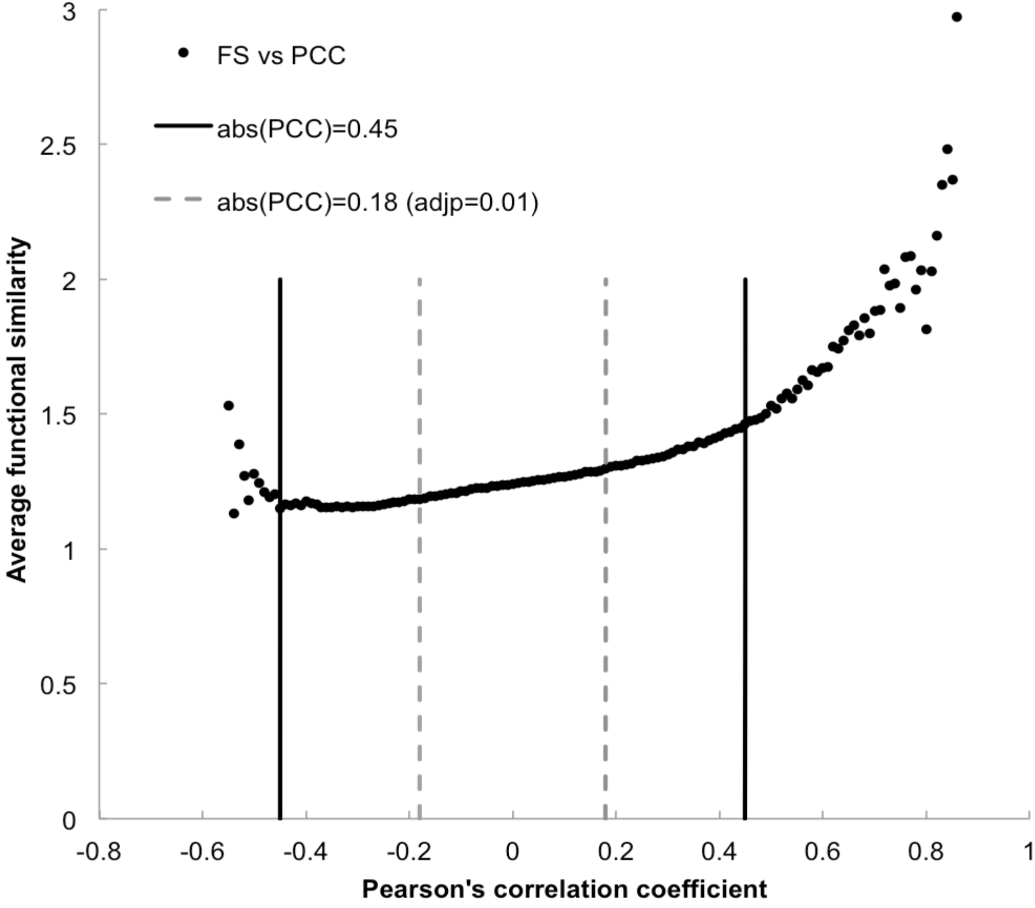

FS vs PCC
abs(PCC)=0.45
abs(PCC)=0.18 (adjp=0.01)
Average functional similarity
Pearson's correlation coefficient
3
2.5
2
1.5
1
0.5
0
-0.8
-0.6
-0.4
-0.2
0
0.2
0.4
0.6
0.8
1

Figure S2

A

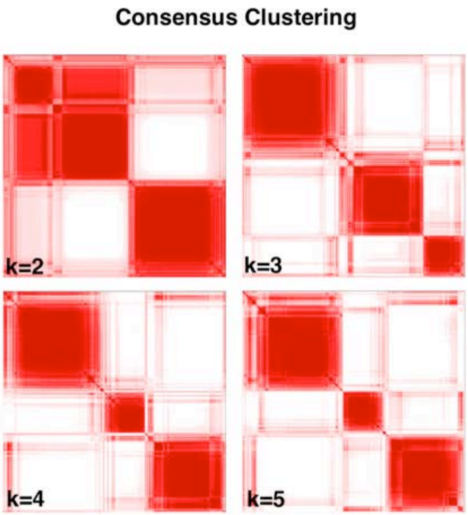

B

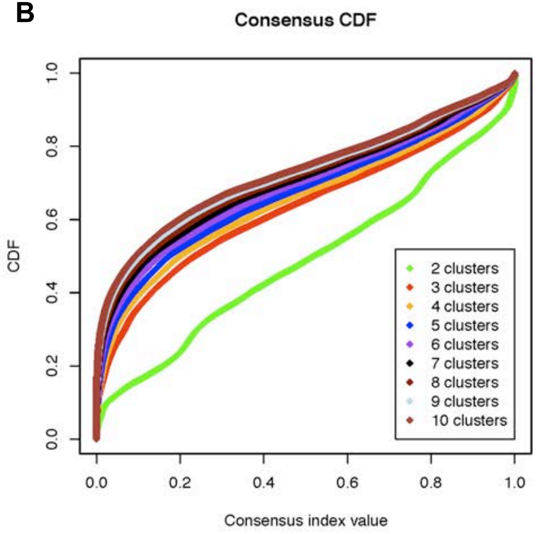

C

Subtype	Subtype_1	Subtype_2	Subtype_3
Subtype_1		< 1.0E-22	< 1.0E-22
Subtype_2			< 1.0E-22
Subtype_3			

D

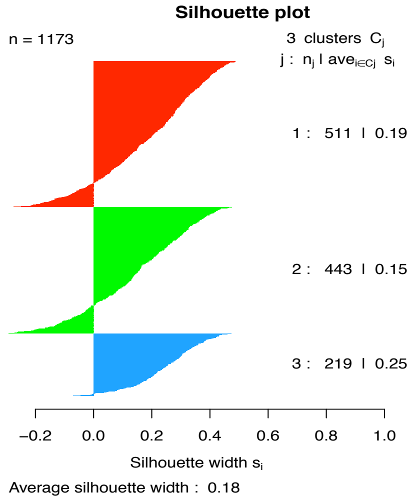

Figure S3

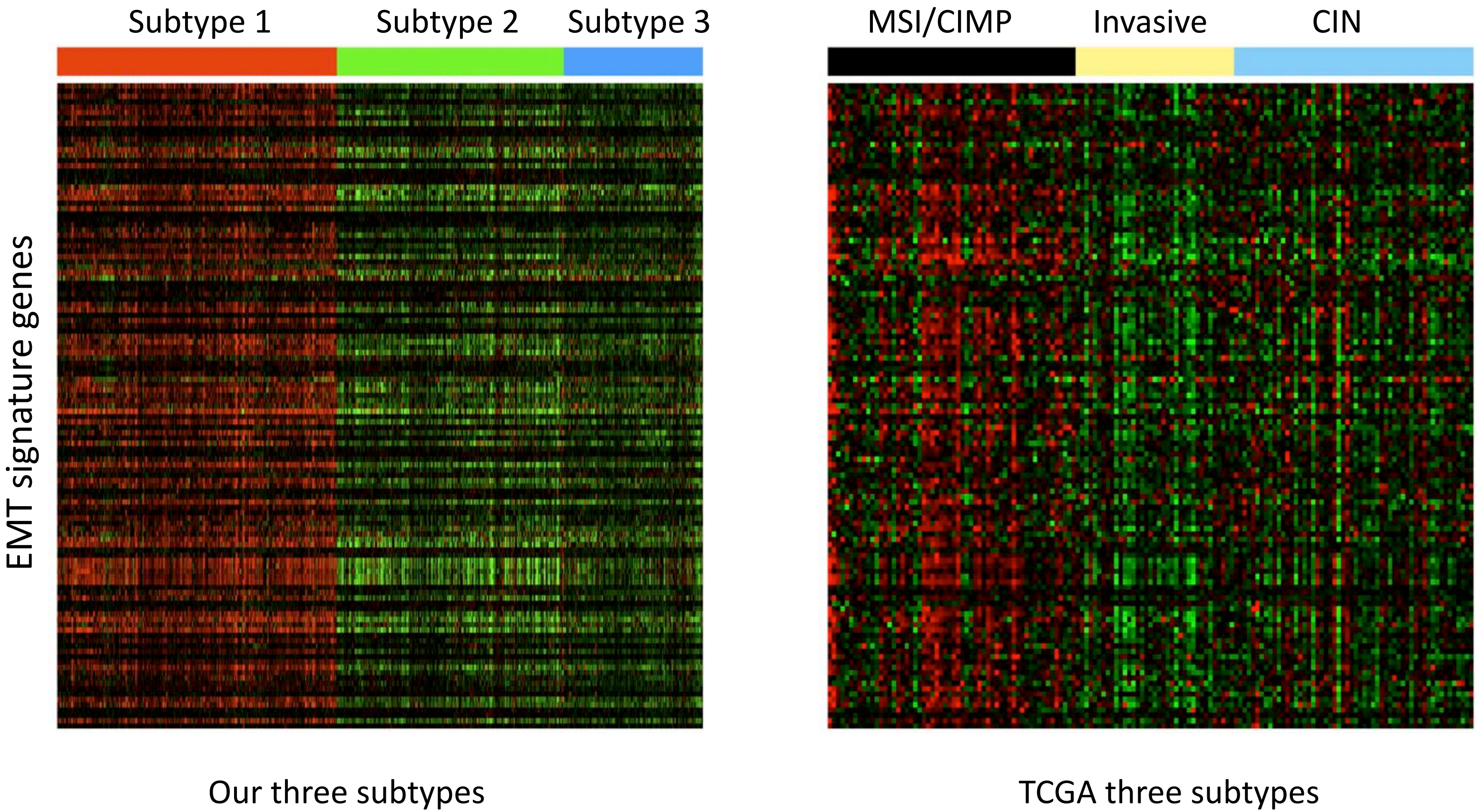
Subtype 1
Subtype 2
Subtype 3
MSI/CIMP
Invasive
CIN
EMT signature genes
Our three subtypes
TCGA three subtypes

Figure S4

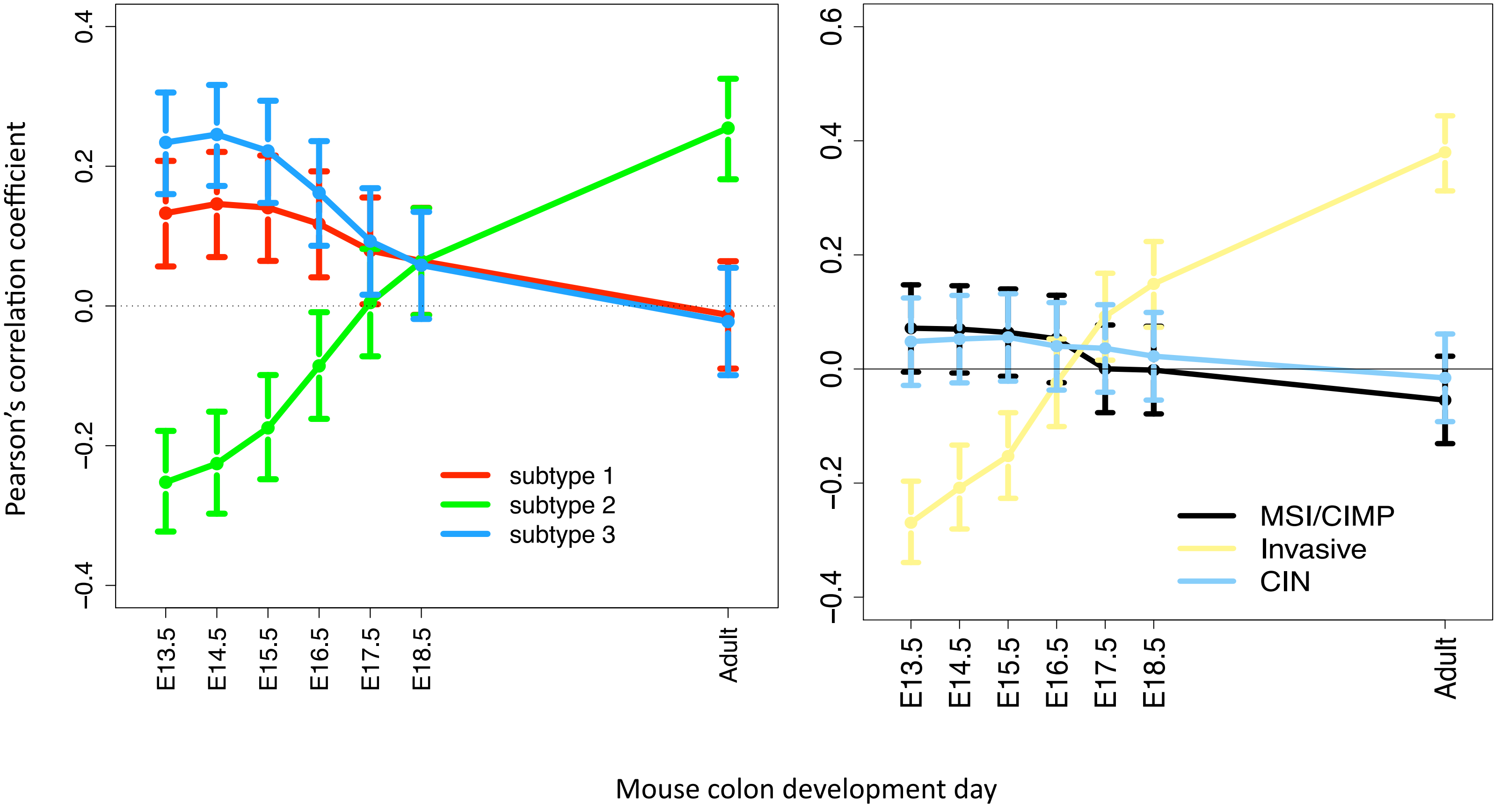

Pearson's correlation coefficient
0.4
0.2
0.0
−0.2
−0.4
subtype 1
subtype 2
subtype 3
E13.5
E14.5
E15.5
E16.5
E17.5
E18.5
Adult
0.6
0.4
0.2
0.0
−0.2
−0.4
MSI/CIMP
Invasive
CIN
E13.5
E14.5
E15.5
E16.5
E17.5
E18.5
Adult
Mouse colon development day

Figure S5

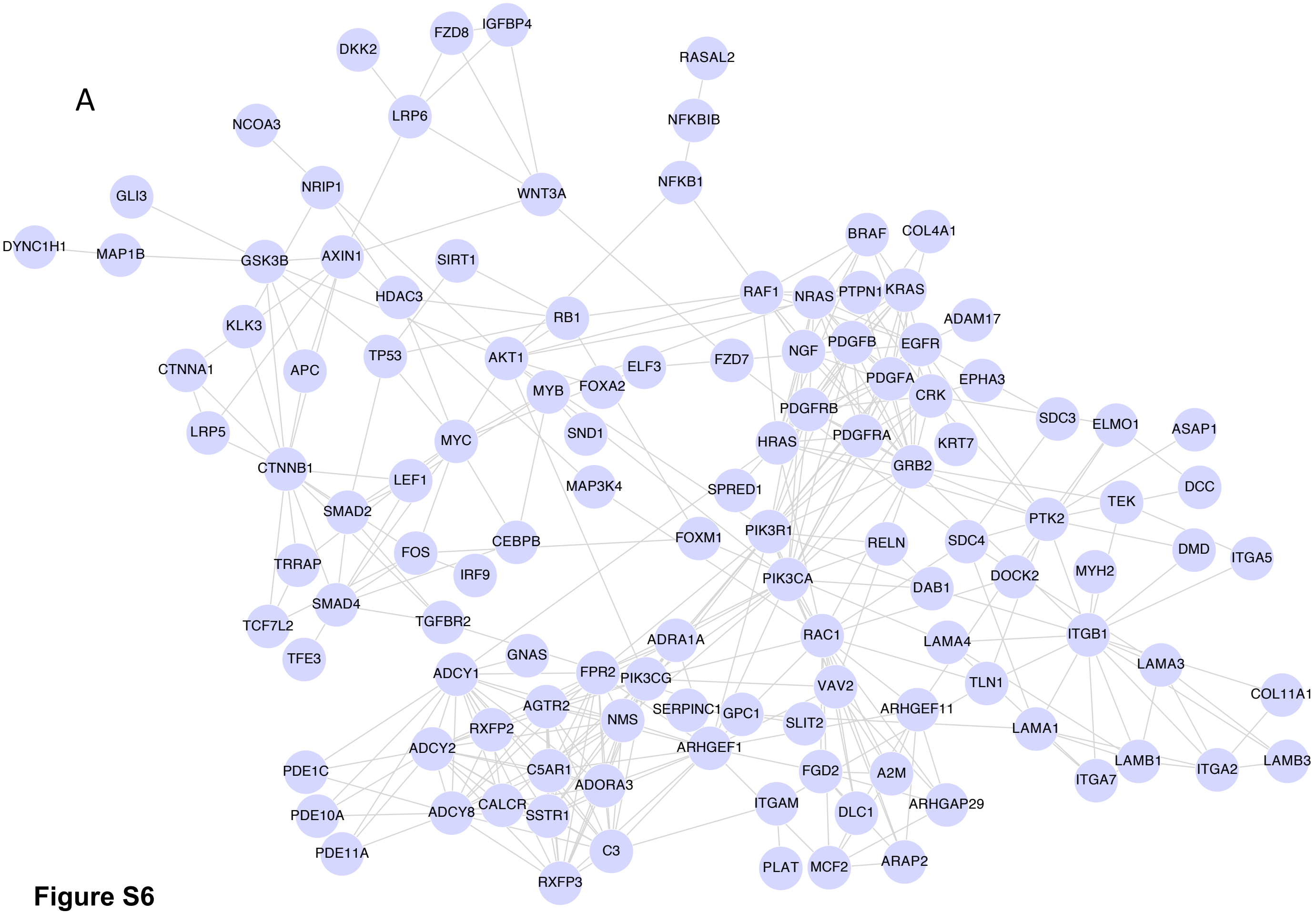

A
IGFBP4
FZD8
DKK2
RASAL2
LRP6
NFKBIB
NCOA3
NRIP1
WNT3A
NFKB1
GLI3
DYNC1H1
MAP1B
GSK3B
AXIN1
SIRT1
BRAF
COL4A1
HDAC3
RAF1
NRAS
PTPN1
KRAS
RB1
ADAM17
KLK3
PDGFB
EGFR
NGF
TP53
AKT1
ELF3
FZD7
CTNNA1
APC
FOXA2
PDGFA
EPHA3
MYB
CRK
PDGFRB
SND1
SDC3
ELMO1
ASAP1
LRP5
MYC
HRAS
PDGFRA
KRT7
CTNNB1
LEF1
MAP3K4
GRB2
DCC
SPRED1
TEK
SMAD2
PTK2
PIK3R1
FOXM1
CEBPB
RELN
SDC4
FOS
DMD
ITGA5
IRF9
TRRAP
PIK3CA
DOCK2
MYH2
DAB1
SMAD4
TGFBR2
TCF7L2
ITGB1
RAC1
LAMA4
ADRA1A
TFE3
GNAS
LAMA3
FPR2
ADCY1
PIK3CG
VAV2
TLN1
COL11A1
SERPINC1
AGTR2
GPC1
ARHGEF11
NMS
RXFP2
SLIT2
LAMA1
ARHGEF1
ADCY2
LAMB1
LAMB3
PDE1C
C5AR1
FGD2
A2M
ITGA2
ITGA7
ADORA3
ITGAM
CALCR
ARHGAP29
PDE10A
ADCY8
SSTR1
DLC1
C3
PDE11A
ARAP2
PLAT
MCF2
RXFP3

Figure S6

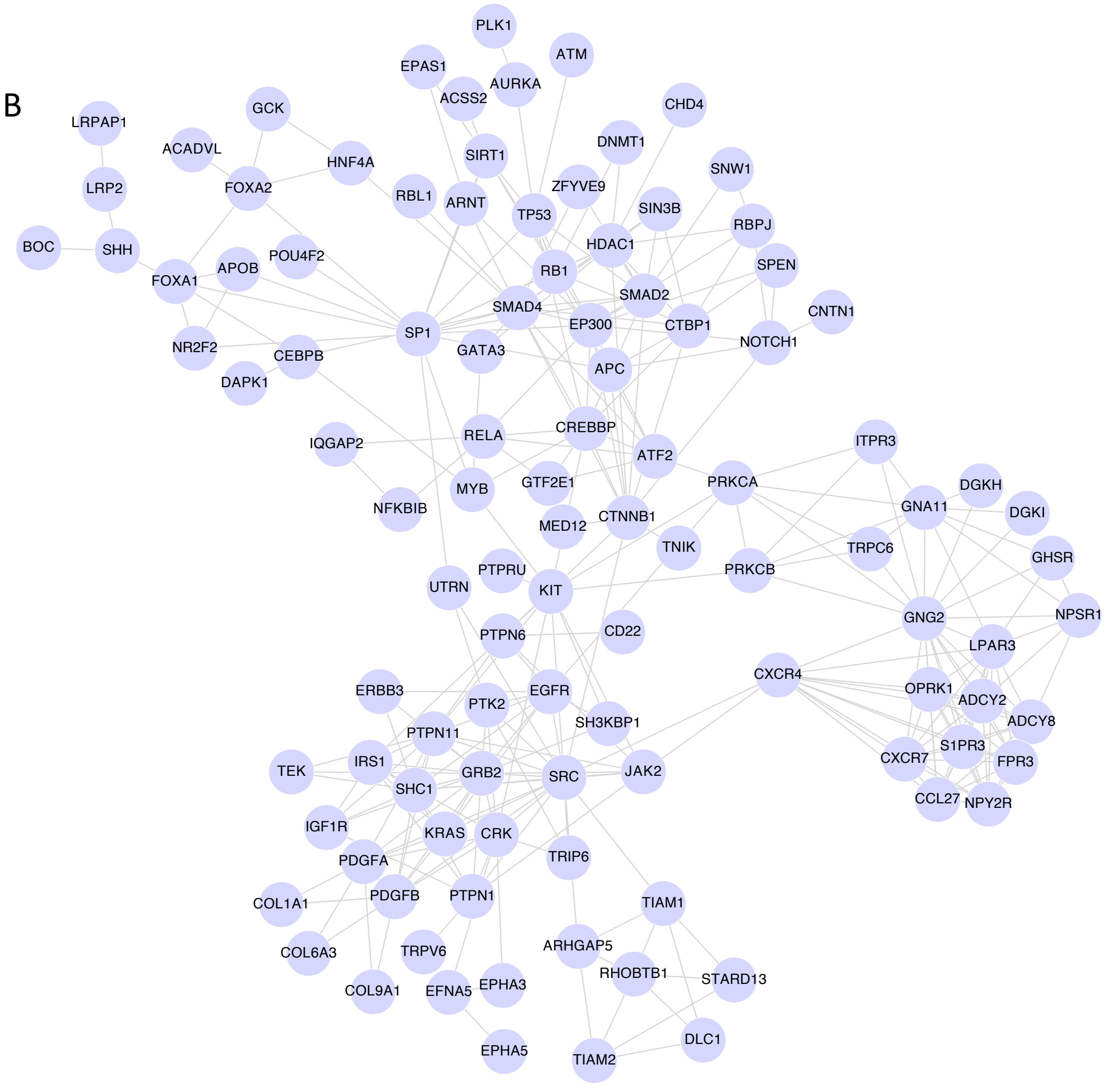
B
PLK1
ATM
EPAS1
AURKA
ACSS2
CHD4
GCK
LRPAP1
DNMT1
ACADVL
SIRT1
HNF4A
SNW1
LRP2
FOXA2
ZFYVE9
RBL1
ARNT
TP53
SIN3B
RBPJ
BOC
SHH
HDAC1
POU4F2
APOB
SPEN
FOXA1
RB1
SMAD2
SMAD4
CNTN1
EP300
CTBP1
SP1
NOTCH1
NR2F2
CEBPB
GATA3
DAPK1
APC
CREBBP
RELA
IQGAP2
ITPR3
ATF2
PRKCA
DGKH
GTF2E1
MYB
NFKBIB
GNA11
DGKI
MED12
CTNNB1
TNIK
TRPC6
GHSR
PTPRU
PRKCB
UTRN
KIT
GNG2
NPSR1
CD22
PTPN6
LPAR3
CXCR4
ERBB3
EGFR
OPRK1
PTK2
ADCY2
SH3KBP1
ADCY8
PTPN11
S1PR3
TEK
IRS1
CXCR7
FPR3
GRB2
JAK2
SRC
SHC1
CCL27
NPY2R
IGF1R
KRAS
CRK
PDGFA
TRIP6
PDGFB
PTPN1
TIAM1
COL1A1
ARHGAP5
COL6A3
TRPV6
RHOBTB1
STARD13
COL9A1
EFNA5
EPHA3
DLC1
EPHA5
TIAM2

Figure S6

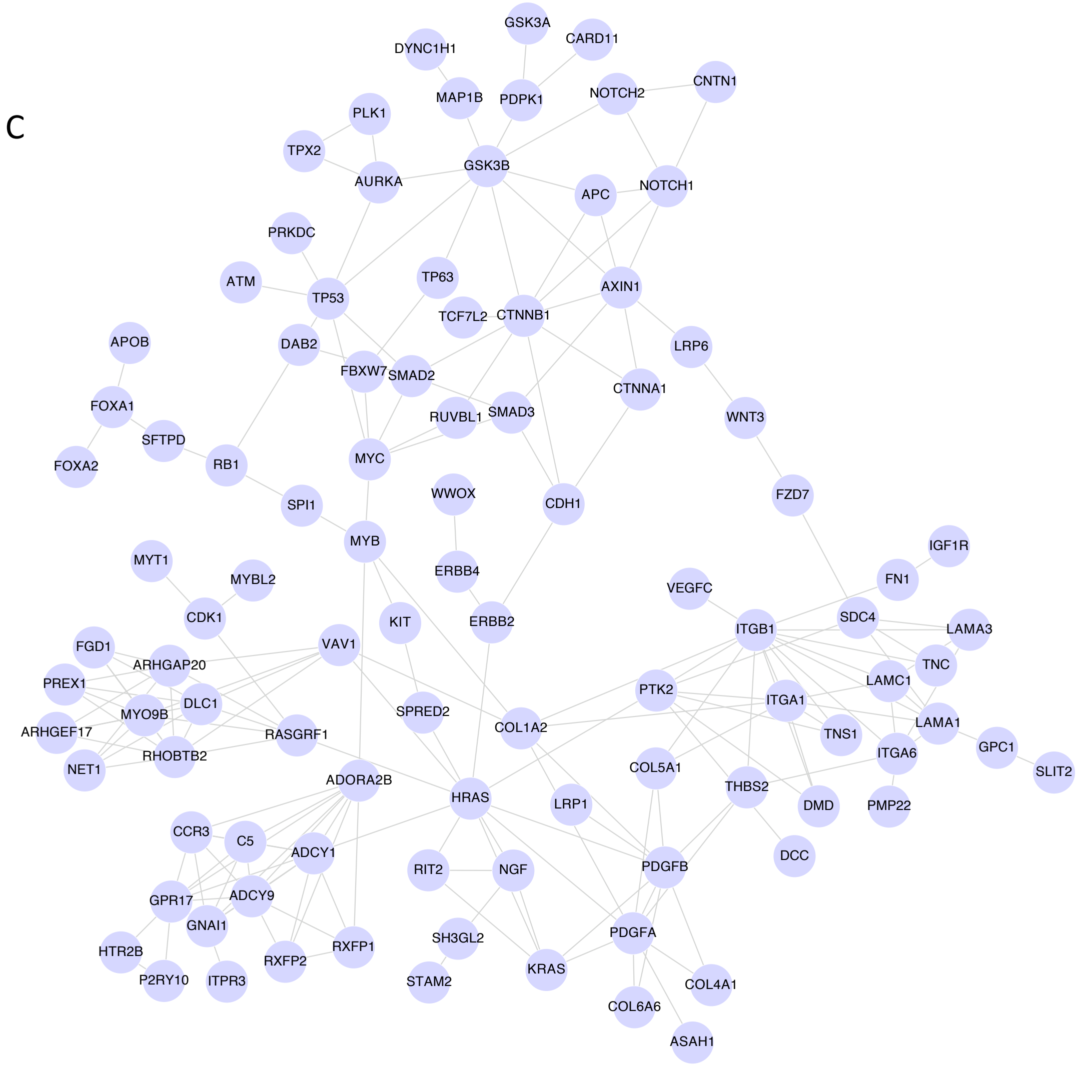
C
GSK3A
CARD11
DYNC1H1
MAP1B
PDPK1
NOTCH2
CNTN1
PLK1
TPX2
AURKA
GSK3B
APC
NOTCH1
PRKDC
ATM
TP53
TP63
TCF7L2
CTNNB1
AXIN1
LRP6
APOB
DAB2
FBXW7
SMAD2
CTNNA1
FOXA1
RUVBL1
SMAD3
WNT3
SFTPD
FOXA2
RB1
MYC
WWOX
CDH1
FZD7
SPI1
MYB
IGF1R
MYT1
MYBL2
ERBB4
VEGFC
FN1
CDK1
KIT
ERBB2
SDC4
ITGB1
LAMA3
FGD1
VAV1
ARHGAP20
TNC
PREX1
DLC1
PTK2
LAMC1
MYO9B
SPRED2
ITGA1
ARHGEF17
RASGRF1
COL1A2
TNS1
LAMA1
GPC1
NET1
RHOBTB2
ITGA6
SLIT2
COL5A1
ADORA2B
HRAS
THBS2
LRP1
DMD
PMP22
CCR3
C5
ADCY1
DCC
RIT2
NGF
PDGFB
GPR17
ADCY9
GNAI1
PDGFA
SH3GL2
RXFP1
HTR2B
RXFP2
KRAS
COL4A1
P2RY10
ITPR3
STAM2
COL6A6
ASAH1

Figure S6

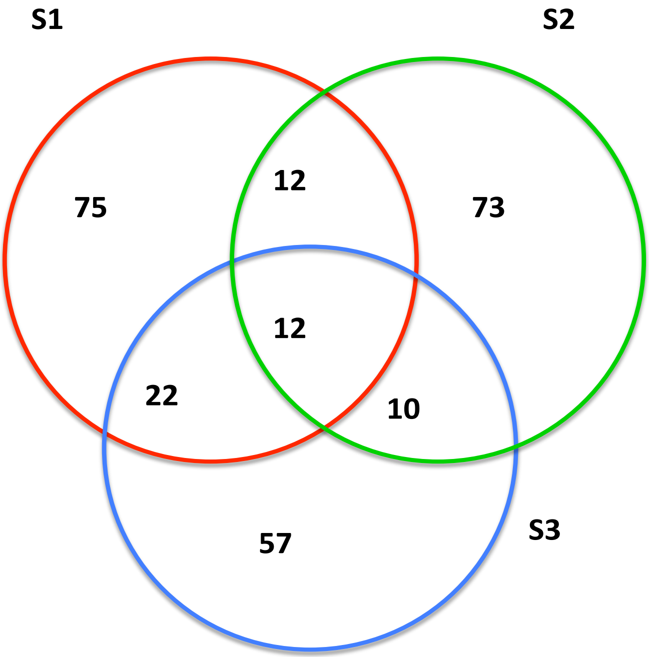

Figure S7

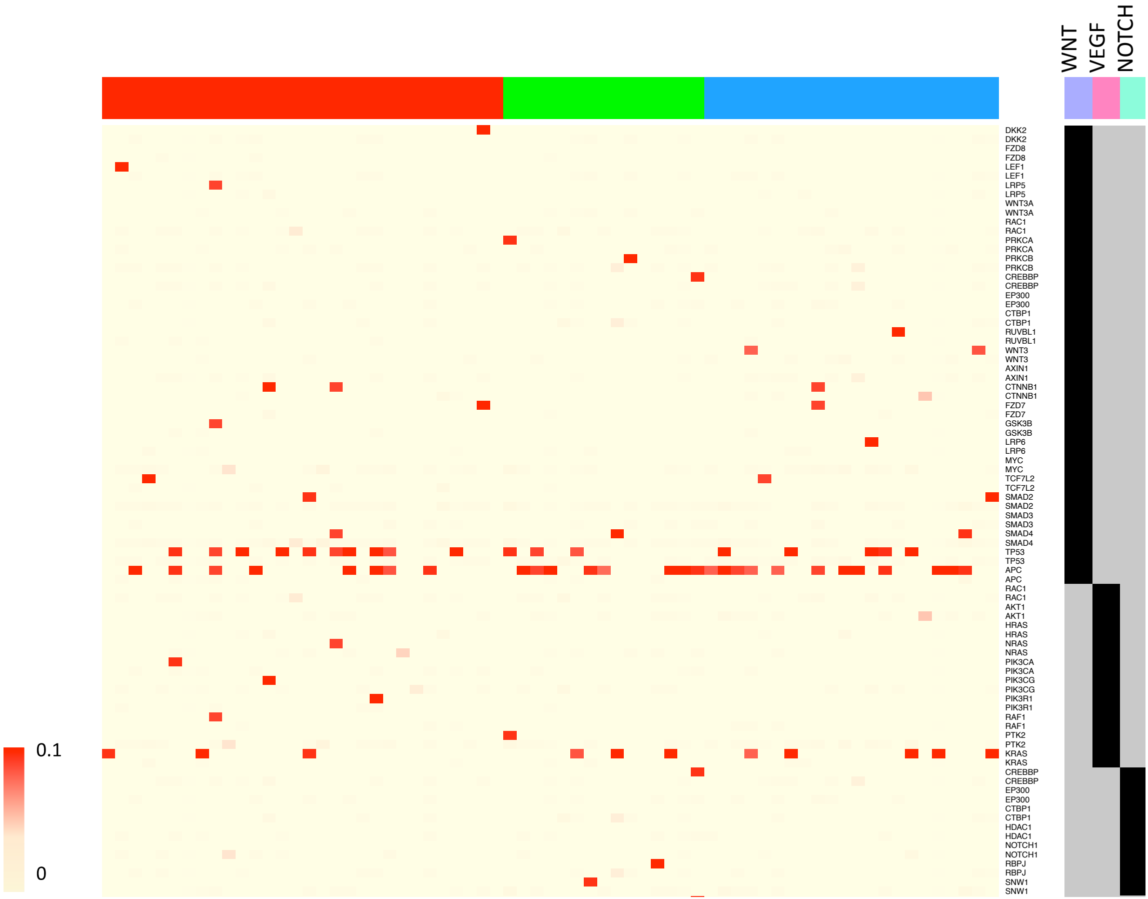
WNT
VEGF
NOTCH
0.1
0
DKK2
DKK2
FZD8
FZD8
LEF1
LEF1
LRP5
LRP5
WNT3A
WNT3A
RAC1
RAC1
PRKCA
PRKCA
PRKCB
PRKCB
CREBBP
CREBBP
EP300
EP300
CTBP1
CTBP1
RUVBL1
RUVBL1
WNT3
WNT3
AXIN1
AXIN1
CTNNB1
CTNNB1
FZD7
FZD7
GSK3B
GSK3B
LRP6
LRP6
MYC
MYC
TCF7L2
TCF7L2
SMAD2
SMAD2
SMAD3
SMAD3
SMAD4
SMAD4
TP53
TP53
APC
APC
RAC1
RAC1
AKT1
AKT1
HRAS
HRAS
NRAS
NRAS
PIK3CA
PIK3CA
PIK3CG
PIK3CG
PIK3R1
PIK3R1
RAF1
RAF1
PTK2
PTK2
KRAS
KRAS
CREBBP
CREBBP
EP300
EP300
CTBP1
CTBP1
HDAC1
HDAC1
NOTCH1
NOTCH1
RBPJ
RBPJ
SNW1
SNW1

Figure S8
